# A hedgehog cathelicidin-derived peptide exhibits antiviral activity against herpes simplex virus type 1 infection

**DOI:** 10.3389/fmicb.2026.1770133

**Published:** 2026-02-18

**Authors:** Enjie Deng, Yaping Pei, Kun Yuan, Juan Yang, Suncheng-ai Cao, Xinyan Yang, Guilan Li, Libin Liang, Lin Jin, Tengyu Zhu

**Affiliations:** 1 Shanxi Key Laboratory for Modernization of TCVM, College of Veterinary Medicine, Shanxi Agricultural University, Taiyuan, China; 2 Department of Anesthesiology, First Affiliated Hospital of Kunming Medical University, Kunming, China

**Keywords:** antiviral activity, CathEE-2a, drug candidate, hedgehog cathelicidin, HSV-1, type I interferons

## Abstract

Herpes simplex virus type 1 (HSV-1) is a widespread infectious virus that poses a substantial public health burden. With no specific curative therapy available and current antivirals providing only symptomatic relief, the development of new effective antiviral agents remains imperative. Antimicrobial peptides, particularly cathelicidins, are key immune effector molecules with antiviral and immunomodulatory properties. In this study, we found that European hedgehog skin extract (HSE) exhibited potent anti-HSV-1 activity, prompting us to investigate the contribution of the cathelicidin peptide CathEE. Based on this observation, three CathEE-derived antiviral peptides (CathEE-1, CathEE-2, and CathEE-3) were designed and further optimized to yield CathEE-2a and CathEE-2b. Notably, CathEE-2a displayed pronounced antiviral activity *in vitro* and significantly reduced brain viral loads in an HSV-1 mouse infection model. Histopathological analyses further revealed that CathEE-2a attenuated HSV-1-induced brain damage. Mechanistically, CathEE-2a suppressed HSV-1 infection by upregulating type I interferons and downstream antiviral genes. Collectively, these findings identify CathEE-2a as an immunomodulatory antiviral peptide and a promising lead candidate for the development of novel therapeutics against HSV-1 infection, highlighting the therapeutic potential of hedgehog-derived antimicrobial peptides in antiviral drug discovery.

## 1 Introduction

Herpes simplex virus type 1 (HSV-1) is an enveloped double-stranded DNA virus primarily transmitted through fluids or droplets derived from cutaneous or mucosal lesions (James et al., 2020). As a clinically important human pathogen, HSV-1 infects an estimated two-thirds of the global population, representing a substantial public health burden. HSV-1 causes a broad range of diseases across the lifespan—from orolabial lesions and ocular keratitis to life-threatening neonatal disseminated infections and adult encephalitis—and is capable of establishing lifelong latency within sensory ganglia, from which it can undergo periodic reactivation and recurrent shedding (Zhang et al., 2017; Bhutta et al., 2021; Imafuku, 2023). Beyond these widespread effects, recent clinical evidence indicates that HSV-1 infection markedly elevates the risk of severe maternal complications and adverse neonatal outcomes (Andrievskaya et al., 2022; Xu et al., 2025). These biological features, combined with the high global prevalence of infection, present significant challenges to interrupting HSV-1 transmission. To this end, effective infectious disease control requires synergistic advances in both diagnostics and therapeutics. Rapid and sensitive diagnostic technologies have become increasingly critical for early pathogen detection, outbreak containment, and targeted treatment. Recent progress includes signal-enhanced lateral flow assays and CRISPR-based biosensing platforms, which enable rapid, point-of-care detection of pathogens with high specificity (Chen et al., 2022; Far et al., 2025; Zeng et al., 2025). Standard clinical management relies predominantly on acyclovir (ACV) and its derivatives, which require viral thymidine kinase-mediated phosphorylation and subsequently inhibit viral DNA polymerase to suppress replication (Kukhanova et al., 2014; Aribi Al-Zoobaee et al., 2020; Stoyanova et al., 2023). Although effective in reducing symptom severity, these agents do not eradicate latent virus, enabling persistent infection. Moreover, the emergence of ACV-resistant strains, particularly in immunocompromised patients, further limits therapeutic efficacy (Piret and Boivin, 2011; Stoyanova et al., 2023). Current therapeutic strategies for HSV-1 are constrained by limitations that mirror broader challenges in anti-infective treatment. The prolonged and widespread use of conventional antiviral agents, together with antibiotics in clinical practice, has accelerated the emergence of multidrug resistance, whereas off-target toxicity and inadequate tissue penetration continue to undermine therapeutic efficacy across diverse infectious diseases (Braconi et al., 2025; Yang et al., 2025; Zhuang et al., 2025). These persistent shortcomings have underscored the need for alternative therapeutic approaches and have driven growing interest in the development of agents with novel and mechanistically distinct modes of action.

Antimicrobial peptides (AMPs) are an evolutionarily conserved yet highly diverse class of host-defense molecules and constitute key effectors of innate immunity in vertebrates (Benítez-Prián et al., 2024). Cathelicidins are a major AMP lineage widely distributed among mammals, birds, reptiles, and fish (Scheenstra et al., 2020). Cathelicidins are typically produced as precursor peptides comprising a signal peptide, a conserved N-terminal cathelin domain, and a variable C-terminal mature peptide, and exhibit pronounced structural diversity across species (e.g., *α*-helical, *β*-hairpin, and extended conformations), which is thought to broaden their functional scope and pathogen coverage (Zanetti et al., 1995; Pazgier et al., 2013; Scheenstra et al., 2019). In addition to direct antimicrobial activity, cathelicidins exert pleiotropic immunomodulatory functions, including regulation of epithelial barrier defense, chemotaxis, and inflammatory signaling (Scheenstra et al., 2020; Narh et al., 2024; Yoon et al., 2024). Accumulating evidence indicates that cathelicidins exert antiviral activity through both direct virion disruption and host-directed immune potentiation, including enhancement of type I interferon signaling and antiviral gene expression. For example, the human cathelicidin LL-37 has been reported to inhibit HSV-1 infection by impairing viral entry, and to enhance IFN-*β* production and IRF3 phosphorylation in response to Enterovirus 71 (EV71) and Venezuelan equine encephalomyelitis virus (VEEV) (Ahmed et al., 2019; Yu et al., 2021). Chicken cathelicidin-derived peptides can confer resistance to pseudorabies virus through Toll-like receptor 4 (TLR4) activation (Ye et al., 2023). And the chicken cathelicidin-derived peptide CATH-2 has been shown to promote IL-1β secretion and caspase-1 activation with anti-inflammatory effects (Peng et al., 2022). Together, these findings underscore the translational potential of cathelicidin scaffolds for developing next-generation antivirals with dual virucidal and immunomodulatory activities. Rapid advances in biotechnology now enable the rational design and synthesis of peptides with improved pharmacological properties and reduced adverse effects (Boto et al., 2018), and compared with conventional antibiotics, AMPs generally show a lower propensity to drive resistance (Cebrián et al., 2023). Accordingly, AMPs represent promising lead compounds, and cathelicidin-based scaffolds offer considerable potential for the development of antiviral therapeutics that simultaneously target viruses and modulate host immunity.

*Erinaceus europaeus*, a species of small mammals widely distributed across northern China and the Yangtze River basin, holds dual relevance in biomedicine. In traditional Chinese medicine, Corium Erinacei—the dried skin of *E. europaeus*—is valued for its anti-inflammatory and anti-infective properties, with bioactive peptides considered the principal functional components (Xiahou and Han, 2023). Notably, although hedgehogs serve as reservoirs for multiple zoonotic pathogens, they exhibit remarkable self-healing capacity and can recover from infections such as severe fever with thrombocytopenia syndrome virus (SFTSV), suggesting the presence of potent endogenous broad-spectrum antiviral defense mechanisms (Zhao et al., 2022). Cathelicidins, key effector molecules of innate immunity that are highly expressed in skin and epithelial tissues, have demonstrated activity against a variety of pathogens and represent potential candidates mediating these protective effects. However, the potential antiviral activity of hedgehog-derived cathelicidins against HSV-1 remains unexplored.

In this study, we found that viral infection markedly upregulated cathelicidin expression in hedgehog skin, and that exogenous supplementation of cathelicidin conferred antiviral activity *in vitro*. To improve antiviral potency, we rationally designed, optimized, and synthesized an 18-amino acid derivative, CathEE-2a. Subsequent analyses demonstrated that CathEE-2a exerted post-entry antiviral activity and enhanced host antiviral immunity through activation of the type I interferon pathway. Moreover, in an HSV-1 mouse infection model, CathEE-2a significantly reduced brain viral loads and attenuated virus-induced histopathological damage. Collectively, these findings identify CathEE-2a as a promising lead candidate for the development of antimicrobial peptide-derived antiviral therapeutics.

## 2 Materials and methods

### 2.1 Cells and virus

U251 cells (human glioma cell line), Vero cells (African green monkey kidney epithelial cell line) and HSF cells (hedgehog fibroblast cells) were maintained in our laboratory. U251 and Vero cells were cultured in Dulbecco’s Modified Eagle Medium (DMEM; Gibco, Waltham, MA, USA) supplemented with 10% (v/v) heat-inactivated fetal bovine serum (FBS; ExCell Bio, Suzhou, China), 100 U/mL penicillin, and 100 μg/mL streptomycin at 37 °C in a humidified incubator with 5% CO₂. The HSF cell dissociation procedure has been described in detail elsewhere (Walmsley et al., 2016). Briefly, hairless ear tissue was collected from adult hedgehogs following anesthesia with 4% isoflurane. The tissue was minced and digested in a solution of collagenase type I (20 mg/mL, Gibco, Waltham, MA, USA) prepared in 50% complete medium (DMEM supplemented with 50% (v/v) FBS, 1% (v/v) MEM non-essential amino acids (MEM NEAA; Gibco, Waltham, MA, USA), 100 U/mL penicillin, and 100 μg/mL streptomycin, and 1‰ (v/v) 2-Mercaptoethanol (2-ME)) at 37 °C in a thermostatic shaker for 2–3 h. Following digestion, cells were mechanically dissociated by pipetting and filtered through a 70 μm cell strainer (Biosharp, Anhui, China) to obtain a single-cell suspension. The isolated cells were initially cultured in 50% complete medium at 37 °C with 5% CO_2_. After 24–48 h, the medium was replaced with 10% complete medium (DMEM with 10% (v/v) FBS, 1% (v/v) MEM NEAA, 1‰ (v/v) 2-ME, 100 U/mL penicillin, and 100 μg/mL streptomycin), and cells were cultured until reaching sufficient confluence for experimental use.

The HSV-1 (17^+^ strain) used in this study was preserved in our laboratory. Viral stocks were propagated in Vero cells. At 72 h post-infection, culture supernatants were harvested, clarified by centrifugation at 3,000 × g for 10 min at 4 °C, aliquoted, and stored at −80 °C. To ensure stable virulence and titers, the viral stock was subjected to 3 consecutive low-passage propagations. Viral titers were quantified using a standard plaque assay on Vero cells and expressed as plaque-forming units per milliliter (PFU/mL).

### 2.2 Mice

Female C57BL/6 J mice (8 weeks old) were purchased from the Laboratory Animal Center of Shanxi Provincial People’s Hospital (Taiyuan, Shanxi, China). *Ifnar^−/−^* mice were purchased from Cyagen Biosciences Inc. (Guangzhou, Guangdong, China). The mice were group-housed in a controlled environment with a temperature of 22 ± 1 °C and a 12-h light/dark cycle, with free access to autoclaved water and standard rodent chow. All animal care and experiments were approved by the Animal Care Committee of Shanxi Agricultural University (Approval number: SXAU-EAW-2025 M&Rr. BQ.004015379).

### 2.3 Evaluation of anti-HSV-1 activity of the extract from hedgehog skin

Six-month-old female hedgehogs were purchased from Hunan Fulongkang Breeding Co., Ltd. To prepare hedgehog skin extract (HSE), fat-free epidermal tissue was first snap-frozen in liquid nitrogen and ground into a fine powder using a mortar and pestle. The powder was transferred onto ice, homogenized using a Jingxin Auto Sample Grinder-24 L (Shanghai Jingxin Industrial Development Co., Ltd.), and then lysed with 1 mL of ice-cold lysis buffer per portion of powder. The lysis buffer contained 0.5 M Tris–HCl (pH 8.0), 5 mM EDTA (pH 8.0), 150 mM NaCl, 10 mM MgCl₂, 2% (v/v) 2-ME and a protease inhibitor cocktail. The lysate was incubated at 4 °C for 30 min with gentle rotation to ensure complete lysis and subsequently centrifuged at 12,000 rpm for 15 min at 4 °C. The supernatant was carefully harvested, avoiding both the upper lipid layer and bottom insoluble pellet, to obtain a crude protein extract (Yum et al., 2017). To remove endotoxin, the crude extract was dialyzed at 4 °C using an endotoxin-removing dialysis bag with a molecular weight cutoff of 10 kDa, with endotoxin-free PBS as the external dialysis buffer. The external buffer was replaced three times at appropriate intervals to thoroughly eliminate residual detergents, salts, and endotoxins. Following protein concentration determination via the BCA assay, anti-HSV-1 activity was evaluated by seeding U251 cells in 12-well plates at a density of 1.5 × 10^5^ cells/well and culturing them for 12 h. Cells were then infected with HSV-1 at a multiplicity of infection (MOI) of 0.05 and then immediately treated with hedgehog skin extract (25 or 50 μg/mL) or acyclovir (ACV, 5 μg/mL) for 24 h. Finally, total cellular DNA was extracted and analyzed by quantitative PCR (qPCR).

### 2.4 Analysis of HSV-1 infection on hedgehog *CAMP* gene expression

Hedgehog fibroblast cells were seeded in 12-well plates at a density of 2 × 10^5^ cells per well and cultured for 12 h at 37 °C with 5% CO₂. The cells were then infected with HSV-1 at an MOI of 0.1 for 1 h at 37 °C. After infection, the inoculum was replaced with virus maintenance medium, and incubation continued for 24 h. A blank control group (designated as Vehicle) was included in the experiment. Total RNA was then isolated and subjected to Quantitative RT-PCR (RT-qPCR) analysis.

### 2.5 Peptide design and synthesis

The cathelicidin sequence of *E. europaeus* was identified through keyword searches (“*Erinaceus europaeus*” and “cathelicidin”) in the National Center for Biotechnology Information (NCBI; https://www.ncbi.nlm.nih.gov/). The mature peptide sequence was designated as CathEE. By truncating the template peptide to retain its *α*-helical functional region, three modified peptides CathEE-1, CathEE-2 and CathEE-3 were obtained. Following the initial screening, the most potent anti-HSV-1 peptide was chosen as the template for rational design. Specific amino acid substitutions and insertions were introduced, resulting in two derivatives designated CathEE-2a and CathEE-2b. Specifically, the peptide was engineered by replacing neutral/polar residues with arginine (Arg) and lysine (Lys) to increase net positive charge, thereby enhancing antimicrobial activity while minimizing toxicity. The proportion of hydrophobic residues was adjusted to approximately 40–60%, thereby enhancing selectivity. Notably, during these modifications, the functional α-helical conformation was preserved, with the polar/nonpolar residue ratio maintained at roughly 1:1. The C-termini of all the peptides were amidated (-NH2). These peptides were chemically synthesized by Gill Biochemical Co., Ltd. (Shanghai, China), with purity exceeding 95% as verified by reversed-phase high-performance liquid chromatography (HPLC) and mass spectrometric (MS) characterization (Supplementary Table 1).

### 2.6 Peptide structure prediction and structural analysis

Structural analyses and visualizations of the peptides were performed as follows:

(1) Peptide Structure Prediction: Peptide structures were predicted from the primary amino acid sequences using AlphaFold 3^1^ with its default parameters.
(2) Helical Wheel Projection: Helical wheel projections (Schiffer–Edmundson diagrams) were generated using the HeliQuest server.^2^ Analyses were performed using the full-length sequence of each peptide (window size: full) with default parameters: a rotation angle of 100°, a projection radius of 2.5 Å per residue, and 3.6 residues per turn. Residues were color-coded as follows: yellow for nonpolar hydrophobic residues, blue for polar basic residues, red for negatively charged residues, pink for amine-containing residues, purple for hydroxy amino acids, and gray for glycine (Gly).
(3) Three-Dimensional Structure Visualization: Three-dimensional structures were visualized and rendered using PyMOL 3.1.1 (Schrödinger, LLC). Secondary structures were displayed in cartoon representation with RGB color coding: *α*-helices in red (1.0, 0.2, 0.2), turns in green (0.2, 0.8, 0.2), and coils in gray (0.7, 0.7, 0.7).
(4) Electrostatic Surface Potential Analysis: Molecular surfaces and electrostatic potentials were calculated and visualized in PyMOL. Surfaces were colored according to electrostatic potential (blue for positive and red for negative) with a transparency of 0.15 and overlaid on stick representations. Specific residue colors were applied as follows: basic residues RGB (0.6, 0.6, 0.9) and acidic residues RGB (0.9, 0.5, 0.5). Residues were labeled with one-letter codes followed by residue numbers, with labels offset from the side chain by 3.0 Å.

### 2.7 Cell viability assay

The cytotoxicity of the peptides on U251 cells was assessed using a Cell Counting Kit-8 (CCK-8, Biosharp, Anhui, China). Briefly, U251 cells were seeded into 96-well plates with 1.0 × 10^4^ cells per well for 12 h. Subsequently, cells were exposed to the peptides at concentrations ranging from 0 to 160 μM (0, 1.25, 2.5, 5, 10, 20, 40, 80, and 160 μM). After 24 h treatment, 10 μL CCK-8 reagent was added to each well and incubated for 0.5–4 h at 37 °C. Absorbance was measured at 450 nm using a SpectraMax M2e Multi-Mode Microplate Reader (Molecular Devices, San Jose, CA, USA) with SoftMax Pro 7.0 software.

### 2.8 Assays for antiviral activity assessment *in vitro*

U251 cells were seeded in 12-well plates at a density of 2 × 10^5^ cells per well (1 mL/well) and cultured for 12 h. Then, they were infected with HSV-1 at 0.05 MOI and treated with the peptides (10 μM) or ACV (10 μM; positive control). After incubation for 24 h, total DNA was extracted, and the relative expression levels of HSV-1 *UL30* gene were detected by qPCR.

To assess whether CathEE-2a directly inactivates HSV-1, viral particles were incubated with the indicated concentrations of CathEE-2a at 37 °C for 1, 2, or 4 h. Following incubation, the mixtures were diluted 200-fold to terminate peptide activity. The treated virus was then used to infect U251 cells, and viral replication was evaluated by qPCR analysis of the HSV-1 *UL30* gene, with *UL30* levels normalized to the *18S rDNA* reference gene. A control mixture consisting of HSV-1 and an equivalent amount of CathEE-2a without pre-incubation was included.

To examine whether CathEE-2a interferes with viral attachment, U251 cells were incubated with HSV-1 (MOI = 0.05) in the presence or absence of CathEE-2a at 4 °C for 1 h. After adsorption, unbound virus and peptide were removed by extensive washing, and the amount of cell-associated HSV-1 *UL30* levels was quantified by qPCR and normalized to *18S rDNA*.

To assess the effect of CathEE-2a at the post-entry stage, U251 cells were first infected with HSV-1 (MOI = 0.05) at 37 °C for 1 h to allow viral adsorption and entry. The cells were then treated with different concentrations of CathEE-2a for 24 h. Intracellular HSV-1 *UL30* gene levels were determined by qPCR using *18S rDNA* as the internal reference gene.

### 2.9 Mouse bone marrow–derived macrophages (BMDMs) and HSV-1 infection assay

BMDMs were generated from the tibias and femurs of mice. Isolated bone marrow cells were cultured in DMEM supplemented with 10% (v/v) FBS, 20 ng/mL macrophage colony-stimulating factor (M-CSF; Merck KGaA, Darmstadt, Germany), 100 U/mL penicillin, and 100 μg/mL streptomycin for 7 days at 37 °C under 5% CO₂ to allow differentiation into macrophages.

Differentiated BMDMs from wild-type (WT) and *Ifnar^−/−^* mice were seeded and allowed to adhere overnight. Cells were then infected with HSV-1 at an MOI of 0.1 and simultaneously treated with CathEE-2a at final concentrations of 5 μM (low dose) or 10 μM (high dose). At 12 h post-infection, cells were harvested for total RNA extraction. The mRNA levels of *Ifna*, *Ifnb*, and the interferon-stimulated genes *Isg15* and *Mx1* were quantified by RT-qPCR, with *Hprt* as the internal reference gene. At 24 h post-infection, culture supernatants were collected for viral titration by standard plaque assay on Vero cells. In parallel, total cellular DNA was extracted, and HSV-1 *UL30* gene levels were quantified by qPCR and normalized to *18S rDNA*.

### 2.10 Isolation RNA and DNA for RT-qPCR and qPCR analyses

Total RNA was isolated using TRIzol reagent (Invitrogen, Waltham, MA, USA), and cDNA was reverse-transcribed by using the HiScript III RT SuperMix for qPCR (+ gDNA wiper) kit (Vazyme, Nanjing, China). Total DNA was extracted using TIANamp Genomic DNA Kit (TIANGEN, Beijing, China) according to the manufacturer’s instructions. RNA and DNA purity and concentration were determined using a NanoDrop One UV–Vis Spectrophotometer (Thermo Fisher Scientific, Waltham, MA, USA).

RT-qPCR and qPCR were performed on a CFX Connect Real-Time System (Bio-Rad, Hercules, CA, USA) with ChamQ Universal SYBR qPCR Master Mix (Vazyme, Nanjing, China), employing the following thermal cycling protocol: initial denaturation at 95 °C for 5 min, followed by 40 cycles of 95 °C for 10 s and 60 °C for 30 s. The reference genes used were as follows: human *18S rDNA* or *HPRT* for U251 cells, hedgehog *A-tubulin* for hedgehog fibroblasts, and mouse 18S *rDNA* or *Hprt* for BMDMs. Primer sequences are listed in Supplementary Table 2.

### 2.11 Animal experiments

Female C57BL/6 J mice were acclimated for 7 days and randomly assigned into four groups (*n* = 5 per group) using a computer-generated randomization scheme. The groups included a vehicle control group (PBS), an HSV-1-infected control group, an HSV-1 + CathEE-2a (10 mg/kg) treatment group, and an HSV-1 + ACV (10 mg/kg) treatment group. The sample size of 5 mice per group was determined based on our previously established and validated HSV-1 infection model, balancing the need for sufficient statistical power with the ethical principle of minimizing animal use in accordance with the 3Rs guidelines (Yisihaer et al., 2025).

After anesthesia by inhalation of 4% isoflurane, all mice except those in the vehicle control group were subplantarly infected with 1.0 × 10^7^ PFU of HSV-1. This inoculation route and strain combination induces a predictable disease course: local inflammation develops at the injection site at 1–3 days post-infection (dpi), followed by viral dissemination to the spinal cord and brain at 4–6 dpi, which subsequently progresses to hindlimb paralysis and mortality at 7–10 dpi. Mice were euthanized at 24 h after the final treatment, which corresponds to 5 dpi. This time point was chosen to capture the peak of viral dissemination to the central nervous system prior to the onset of severe clinical symptoms, allowing analysis of viral load and host responses during the critical early phase of neuroinvasion (Engel et al., 1997; Jia et al., 2025). At 1 h post-infection, CathEE-2a and ACV were administered daily via intraperitoneal injection at a volume of 100 μL per mouse for 5 consecutive days. The vehicle control group and the HSV-1-infected control group both received an equal volume of PBS intraperitoneally. All treatments were coded by an independent researcher not involved in outcome assessment. Investigators responsible for daily body weight monitoring, sample collection and downstream data analysis remained blinded to group assignments throughout the experiment to minimize observer bias (Verhave et al., 2024). At 24 h after the final treatment, all animals were euthanized by cervical dislocation under deep anesthesia induced with 5% isoflurane. Major tissue samples, particularly the brains, were collected for subsequent analysis.

Following fixation in 4% paraformaldehyde for 72 h, mouse brain tissues were processed for paraffin embedding, sectioning, and staining with hematoxylin and eosin (H&E) staining by Servicebio Biotechnology Co., Ltd. (Wuhan, China).

Mouse brain tissues were homogenized using a tissue grinder, and total DNA was extracted from the homogenate supernatants using the TIANamp Genomic DNA Kit (Tiangen Biotech, Beijing, China) according to the manufacturer’s instructions. Absolute quantification of viral copy numbers was performed using a standard curve-method as previously described (Yisihaer et al., 2025). Briefly, the HSV-1 *UL30* gene was cloned into the pMD19-T vector, and the resulting recombinant plasmids were transformed into *E. coli* DH5α competent cells. Following verification by colony PCR and Sanger sequencing, high-purity plasmid DNA was extracted, quantified using a NanoDrop spectrophotometer, and converted to copy numbers (copies/μL). A 10-fold serial dilution series (10^1^–10^8^ copies/μL) of the plasmid was then used to generate the standard curve. Viral *UL30* gene copy numbers in tissue samples were determined by qPCR using *UL30*-specific primers, with the standard curve for absolute quantification.

### 2.12 Statistical analysis

Figures were generated using the GraphPad Prism 9 (Version 9.00; GraphPad Software, Boston, MA, USA). Data are presented as the mean ± SEM. For analyses involving two independent variables, two-way ANOVA was performed, and for comparisons among three or more groups, one-way ANOVA was employed. Both one-way and two-way ANOVA were followed by Tukey’s post-hoc test to control for family-wise error rate. Normality was assessed using Shapiro–Wilk test and Q-Q plots. A *p* value ≤ 0.05 was considered statistically significant.

## 3 Results

### 3.1 Hedgehog skin extract exerts anti-HSV-1 activity via its active component CathEE

**Figure 1:**
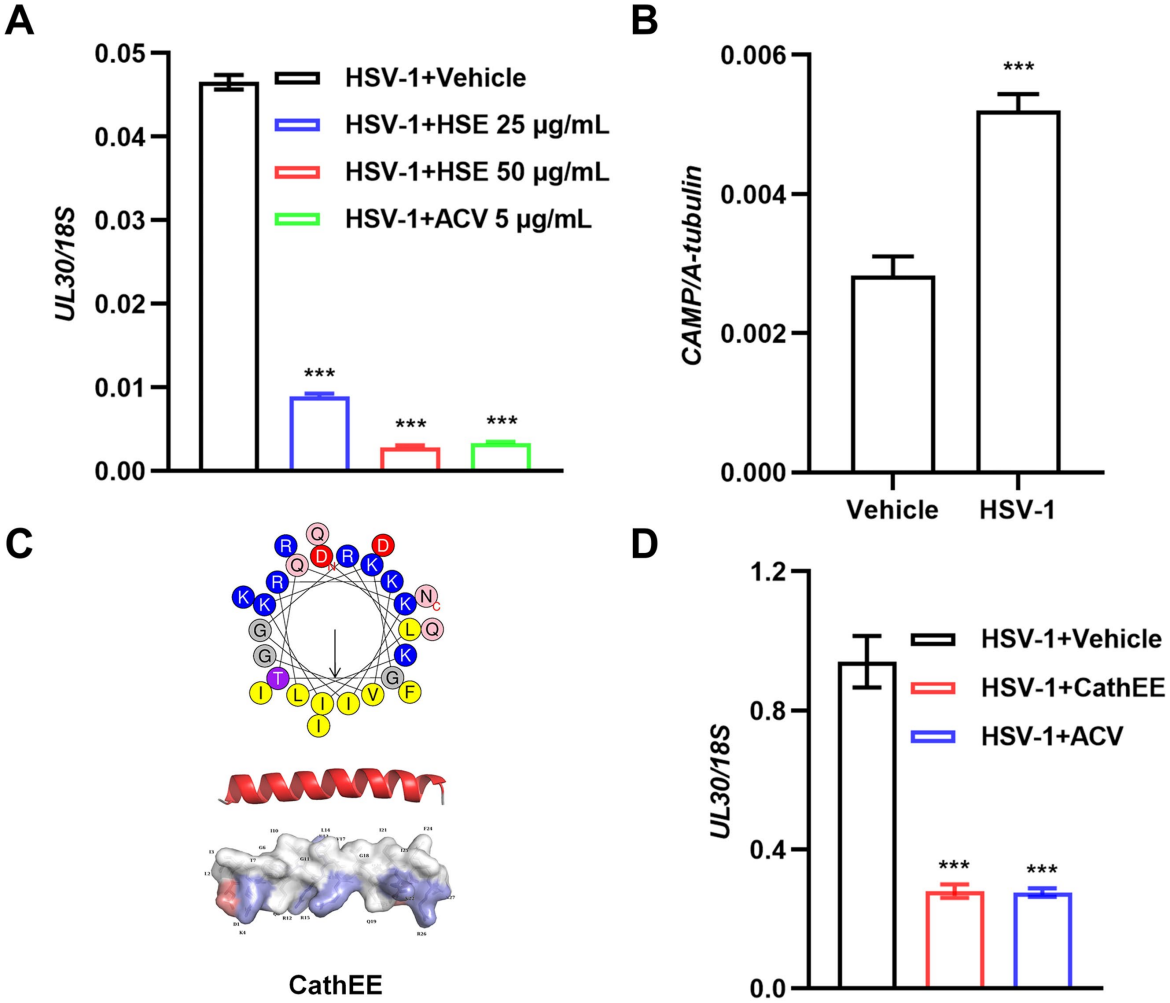
Hedgehog skin extract and its active component CathEE exert anti-HSV-1 activity in U251 cells. **(A)** Antiviral activity of hedgehog skin extract (HSE) against HSV-1 in U251 cells. Cells were infected with HSV-1 (MOI = 0.05) and treated with HSE (25 μg/mL, 50 μg/mL) or acyclovir (ACV, 5 μg/mL; positive control). Viral replication was assessed by qPCR quantification of HSV-1 *UL30* expression (normalized to *18S rDNA*) at 24 h post infection. **(B)** *CAMP* expression levels in HSF cells at 24 h post infection with HSV-1 (MOI = 0.1). Results are presented relative to hedgehog *A-tubulin*. **(C)** Structural features and characterization of CathEE. Upper: Helical wheel projection of CathEE. The C-terminus and N-terminus are labeled “C” and “N,” respectively; residues are color-coded: yellow (nonpolar hydrophobic), blue (polar basic), red (negatively charged), pink (amine-containing), purple (hydroxy amino acids), and gray (glycine). Middle: Amphipathic *α*-helical secondary structure of CathEE. Secondary structure elements are highlighted, with helices in red and coils in white. Lower: Tertiary structure and potential surfaces of CathEE. Surface charge distribution is indicated (blue = positive, red = negative), with potential surfaces of the peptide displayed alongside. **(D)** HSV-1 (MOI = 0.05) was incubated with 10 μM CathEE or the positive control ACV for 24 h, and viral *UL30* was analyzed by qPCR. Results are presented relative to the reference gene *18S rDNA* and normalized to the vehicle group. Data are from 3 independent experiments and are presented as mean ± SEM. Statistical significance was determined by one-way ANOVA followed by Tukey’s post-hoc test for multiple pairwise comparisons **(A,B,D)**. ***p < 0.001.

To investigate the antiviral potential of hedgehog skin extract (HSE), U251 cells were infected with HSV-1 and subsequently treated with HSE at concentrations of 25 μg/mL and 50 μg/mL, with viral replication quantified via quantitative polymerase chain reaction (qPCR). The data revealed that HSE significantly inhibited HSV-1 replication in a dose-dependent manner, and its inhibitory activity was comparable to that of the positive control ACV (5 μg/mL) (Figure 1A). Previous studies have indicated that cathelicidin (abbreviated as CAMP), an endogenous antimicrobial peptide secreted by epithelial cells of humans and other mammals, exhibits broad-spectrum antimicrobial activity and diverse immunomodulatory functions (Bucki et al., 2010). To identify the antiviral active components in HSE, we further assessed the effect of HSV-1 infection on the expression level of *CAMP* in hedgehog skin fibroblast (HSF) cells. Compared with the vehicle-treated group, HSV-1 infection significantly upregulated the relative expression level of *CAMP*. These findings imply that *CAMP*, whose expression is induced by viral infection, may serve as one of the active antiviral components in hedgehog skin (Figure 1B). To further characterize the candidate antiviral component, we identified a mature cathelicidin peptide (designated as CathEE) derived from *E. europaeus* in the NCBI database and analyzed its structural features. CathEE exhibits a typical amphipathic *α*-helical structure (Figure 1C). Subsequent qPCR assays demonstrated that CathEE significantly reduced the *UL30*/*18S* ratio relative to the vehicle-treated HSV-1-infected group, confirming its anti-HSV-1 activity (Figure 1D). Additionally, CCK-8 cytotoxicity assays verified that CathEE showed no cytotoxicity toward U251 cells at all tested concentrations (Supplementary Figure 1).

### 3.2 Structural and functional analysis of truncated CathEE derivatives reveals their anti-HSV-1 activity

**Figure 2:**
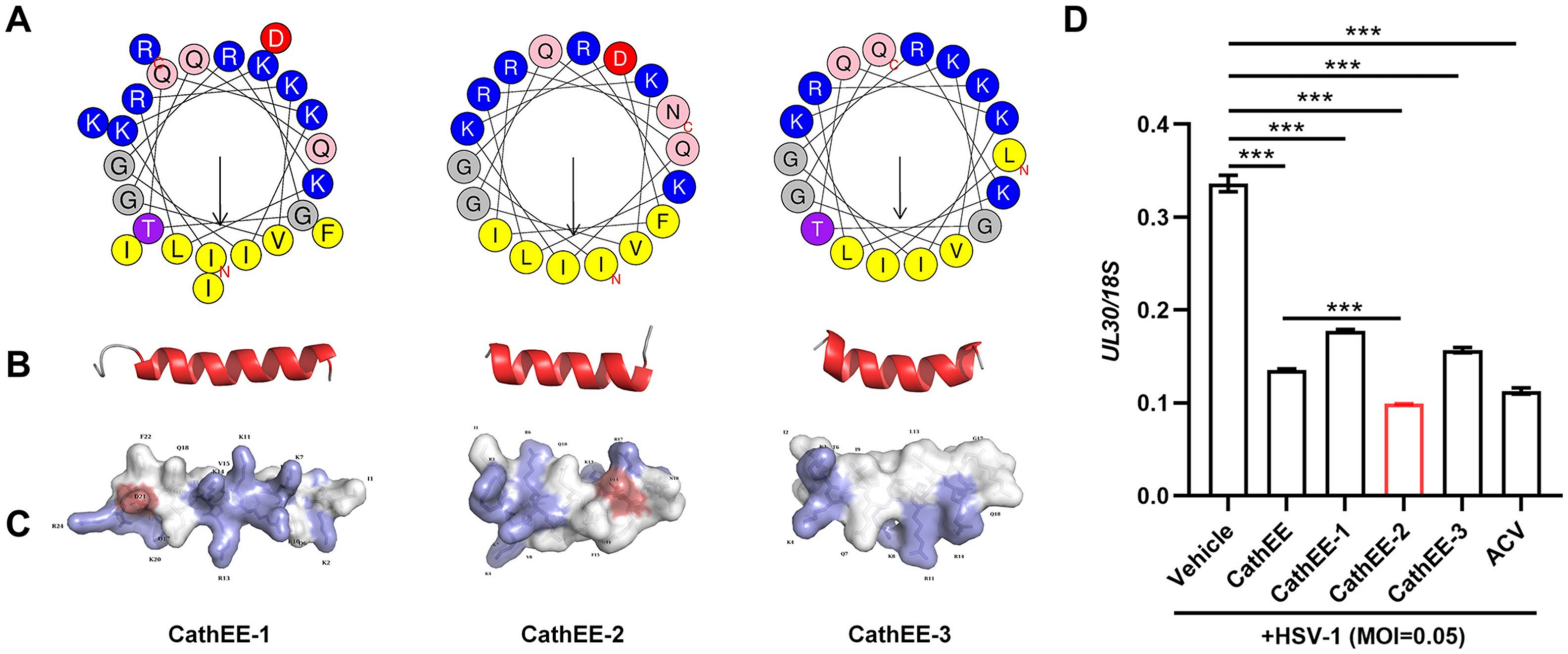
Structural characteristics and anti-HSV-1 activity of truncated CathEE derivatives. **(A)** Schiffer–Edmundson helical wheel projections of CathEE-1, CathEE-2, and CathEE-3. Different color labels represent distinct amino acid residues of the three truncated peptides. **(B)** Secondary structure analysis of CathEE derivatives. All three truncated peptides fold into a typical α-helical conformation, with only minor differences in the flexible terminal regions of the helices. **(C)** Tertiary structure and surface charge distribution of CathEE derivatives. Prominent positively charged regions are marked in blue; CathEE-1 and CathEE-2 exhibit enriched positive charges on their molecular surfaces, whereas CathEE-3 shows a relatively uniform charge distribution. **(D)** Anti-HSV-1 activity of truncated CathEE peptides in U251 cells. Cells were infected with HSV-1 (MOI = 0.05) and treated with CathEE derivatives (10 μM) or ACV (positive control). Viral replication was evaluated by qPCR quantification of the *UL30*/*18S* ratio at 24 h post infection. Data are from 3 independent experiments and are presented as the mean ± SEM. Statistical significance was determined by one-way ANOVA followed by Tukey’s post-hoc test for multiple pairwise comparisons **(D)**. ***p < 0.001.

To pinpoint the minimal functional motif responsible for antiviral activity and optimize the efficacy of the native CathEE peptide, we generated a series of truncated CathEE derivatives and further analyzed their molecular structural characteristics as well as anti-HSV-1 activity. As shown in Figure 2A, Schiffer–Edmundson helical wheel projections revealed that CathEE-1, CathEE-2, and CathEE-3 all adopted an amphipathic α-helical conformation, while distinct differences were observed in their amino acid composition and residue spatial distribution, as indicated by the color-coded residues. Consistent with this observation, secondary structure analysis further confirmed that all three peptides folded into a typical *α*-helical conformation, with only minor differences observed in the flexible regions at the helical terminals (Figure 2B). The surface charge distribution of the tertiary structure demonstrated that prominent positively charged regions (marked in blue) were present on the surfaces of CathEE-1 and CathEE-2, whereas the surface charge distribution of CathEE-3 was relatively uniform (Figure 2C). We next evaluated the antiviral efficacy of these peptides in an HSV-1-infected cell model. Interestingly, all three truncated peptides significantly suppressed HSV-1 replication, among which CathEE-2 showed the strongest inhibitory effect, with activity comparable to that of the positive control ACV, while the inhibitory activities of CathEE-1 and CathEE-3 were slightly weaker but remained statistically significant (Figure 2D). Collectively, these findings indicate that the anti-HSV-1 activity of CathEE peptides is closely related to their structural characteristics, especially the surface charge distribution. Notably, none of the three peptides showed cytotoxicity at the tested concentrations (Supplementary Figure 1).

### 3.3 CathEE-2a exhibits potent anti-HSV-1 activity with low cytotoxicity

**Figure 3:**
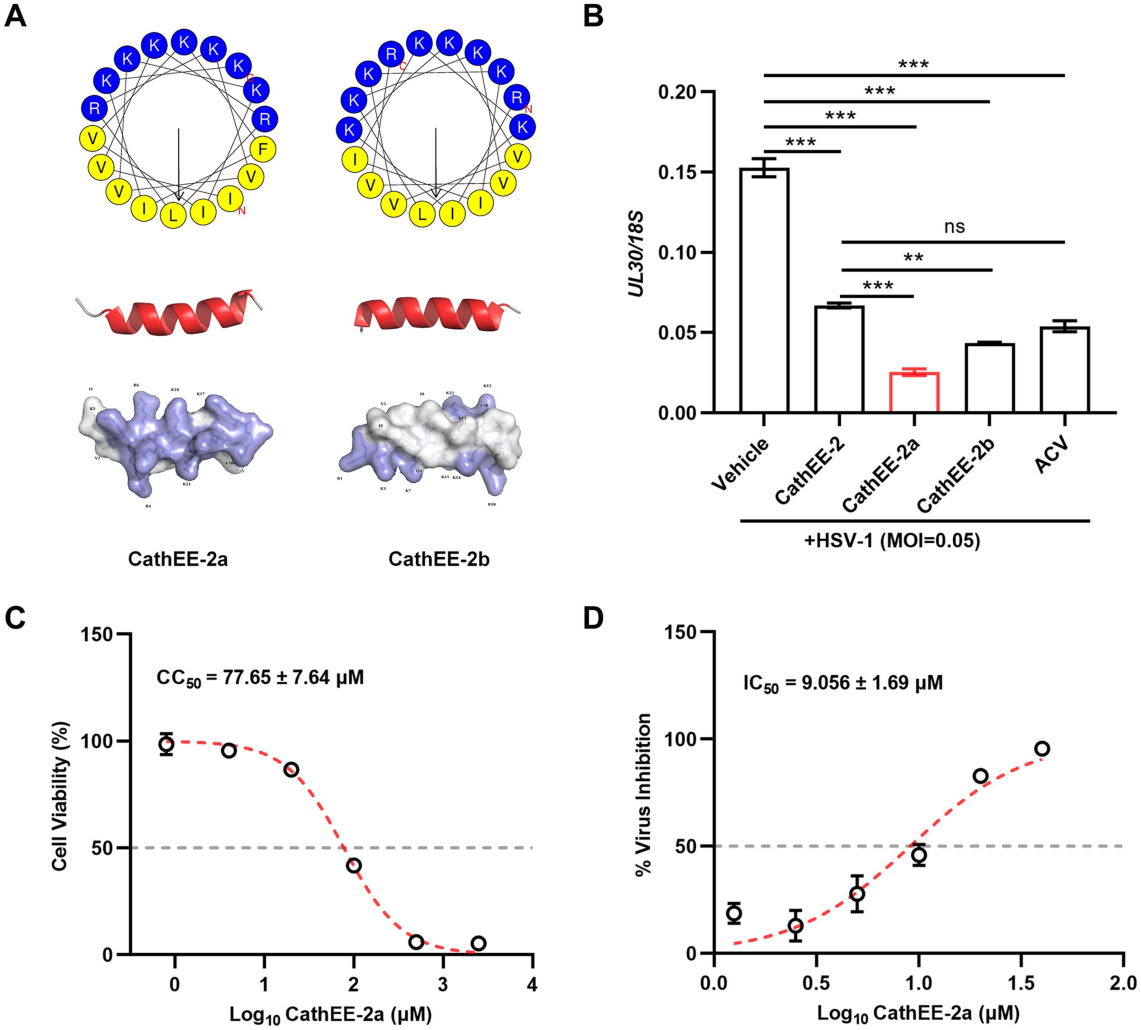
Structural optimization and anti-HSV-1 activity evaluation of CathEE-2 derivatives. **(A)** Structural analyses of CathEE-2a and CathEE-2b, including Schiffer–Edmundson helical wheel projections, predicted three-dimensional structures, and electrostatic potential surfaces. Residues are color-coded by type: yellow (nonpolar hydrophobic) and blue (polar basic). Molecular surface electrostatic potential is shown (blue: positive, red: negative). **(B)** qPCR analysis of HSV-1 replication in U251 cells. Cells were infected with HSV-1 (MOI = 0.05) and treated with 10 μM CathEE-2, CathEE-2a, CathEE-2b, or ACV for 24 h. Results are presented relative to the reference gene *18S rDNA* and normalized to the vehicle group. **(C)** U251 cells were treated with a range of concentrations of CathEE-2a. After 24 h, cell viability was detected via CCK-8 assay, and the CC_50_ was calculated. **(D)** IC_50_ of CathEE-2a against HSV-1. Dose–response curves were used to determine IC_50_ based on viral replication via qPCR. For both panels **(C,D)**: IC_50_ and CC_50_ values were computed using GraphPad Prism 9 software, which applied a four-parameter logistic regression model to the raw, linear (μM) concentration data (not the log-transformed values shown on the axis) to generate the dose–response curves and derive the final values reported in linear μM units. Data are from 3 independent experiments and are presented as the mean ± SEM. Statistical significance was determined by one-way ANOVA followed by Tukey’s post-hoc test for multiple pairwise comparisons **(B)**. ***p < 0.001; ns, not significant.

To further optimize the active motif of CathEE-2, we generated two derivatives (CathEE-2a and CathEE-2b) and characterized their structure and antiviral activity. As shown in Figure 3A, both peptides retained the α-helical secondary structure of the parent peptide, but differed in residue composition and surface charge distribution. Notably, CathEE-2a exhibited more concentrated positive charges on the surface of its predicted three-dimensional structure.

We next evaluated their anti-HSV-1 efficacy in HSV-1-infected U251 cells (MOI = 0.05). CathEE-2a treatment led to a more significant reduction in the *UL30*/*18S* ratio, while the inhibitory effect of CathEE-2b was relatively weak, indicating that structural modification of CathEE-2a enhanced its antiviral activity (Figure 3B). The CCK-8-based cytotoxicity assay showed that CathEE-2a had a high 50% cytotoxic concentration (CC₅₀ = 77.65 ± 7.64 μM), confirming no obvious cytotoxicity at its effective antiviral concentration (Figure 3C). We further determined the half-maximal inhibitory concentration (IC₅₀) of CathEE-2a. The dose–response curve showed that CathEE-2a inhibited HSV-1 replication in a concentration-dependent manner, with an IC₅₀ of 9.056 ± 1.69 μM (Figure 3D). The selectivity index (SI) of CathEE-2a, calculated as the ratio of CC₅₀ to IC₅₀, was ~8.57, reflecting a favorable therapeutic window for anti-HSV-1 treatment. A high SI value indicates that CathEE-2a exerts potent antiviral effects at concentrations far below those that induce host cell cytotoxicity, highlighting its potential as a safe and effective anti-HSV-1 candidate.

### 3.4 CathEE-2a upregulates type I interferon and downstream antiviral genes expression

**Figure 4:**
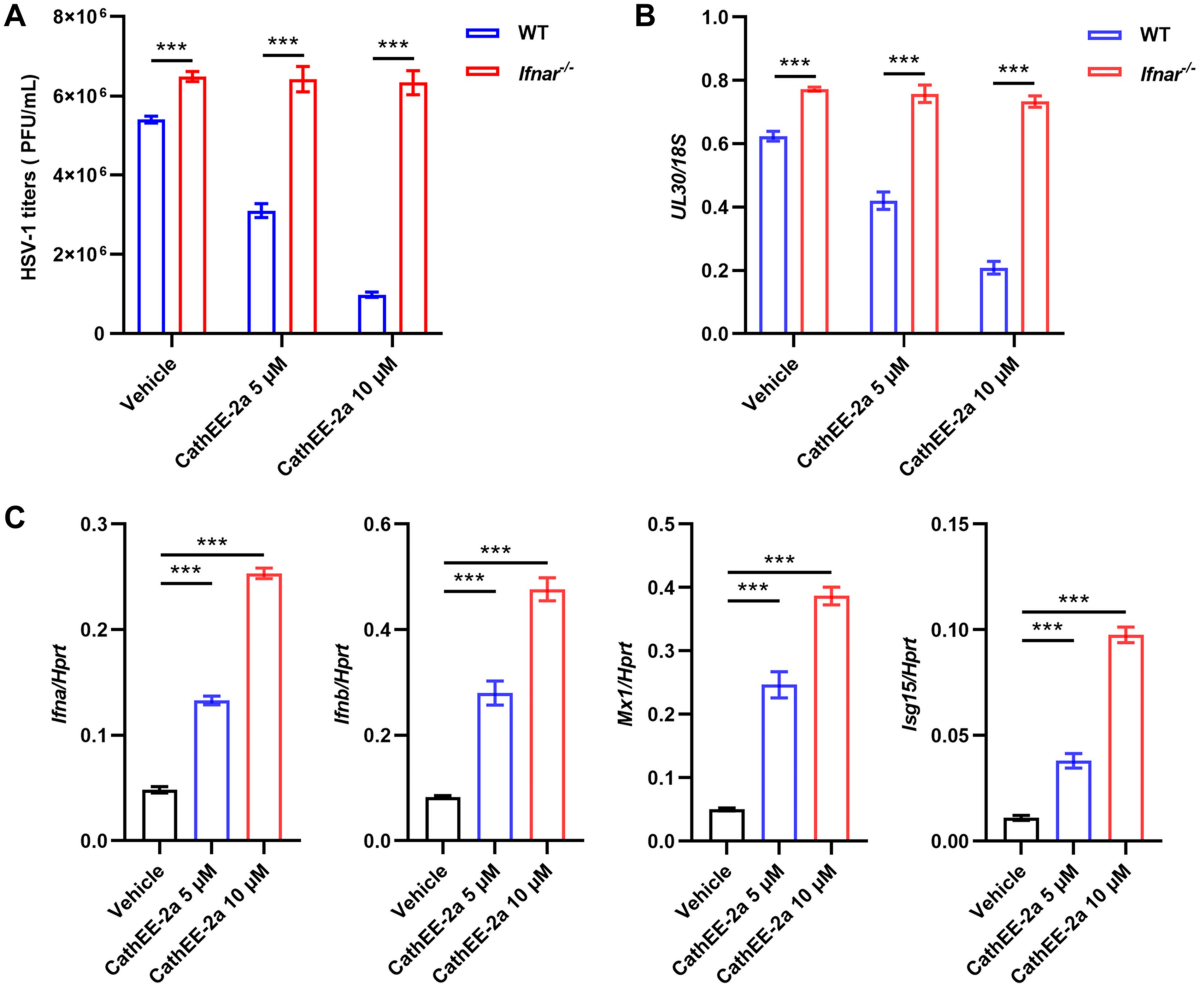
CathEE-2a inhibits HSV-1 infection through a type I IFN-dependent mechanism. **(A)** Bone marrow–derived macrophages (BMDMs) from WT and *Ifnar^−/−^* mice were infected with HSV-1 (MOI = 0.1) and treated with the indicated concentrations of CathEE-2a or vehicle control. At 24 h post-infection, culture supernatants were collected, serially diluted, and subjected to plaque assays. **(B)** HSV-1 *UL30* gene levels in BMDMs under the same experimental conditions as in **(A)**, as determined by qPCR with normalization to the *18S rDNA* reference gene. **(C)** RT-qPCR analysis of *Ifna, Ifnb, Isg15, and Mx1* expression at 12 h post HSV-1 infection in WT BMDMs. Gene expression levels were normalized to the reference gene *Hprt*. Data are from 3 independent experiments and are presented as mean ± SEM. Statistical significance was determined by two-way ANOVA with Tukey’s post-hoc test for multiple pairwise comparisons **(A–C)**. ***p < 0.001.

Following the identification of the post-entry stage as the primary mode of CathEE-2a antiviral activity (Supplementary Figure 2), we subsequently assessed whether CathEE-2a modulates host antiviral immune responses. Analysis in U251 cells indicated that CathEE-2a upregulated key components of the type I IFN signaling pathway (Supplementary Figure 3), suggesting a potential immunomodulatory mechanism. To determine whether type I IFN pathway is functionally required for the antiviral activity of CathEE-2a, we performed antiviral assays using BMDMs from WT and *Ifnar^−/−^* mice. In WT BMDMs, CathEE-2a inhibited HSV-1 infection in a dose-dependent manner, with 10 μM exerting a stronger inhibitory effect than 5 μM. In contrast, the antiviral effect was largely abolished in *Ifnar^−/−^* BMDMs at both concentrations (Figures 4A,B), indicating that CathEE-2a requires intact type I IFN signaling for its antiviral activity. To further delineate this mechanism, we assessed the expression of type I IFNs and downstream effector genes. CathEE-2a treatment increased the mRNA levels of *Ifna*, *Ifnb*, *Isg15*, and *Mx1* in WT BMDMs in a dose-dependent manner at 12 h post-infection (Figure 4C). Collectively, these findings demonstrate that the anti-HSV-1 activity of CathEE-2a is primarily mediated through canonical type I IFN signaling and exhibits clear dose dependence.

### 3.5 CathEE-2a exerts potent *in vivo* therapeutic effects against HSV-1

**Figure 5:**
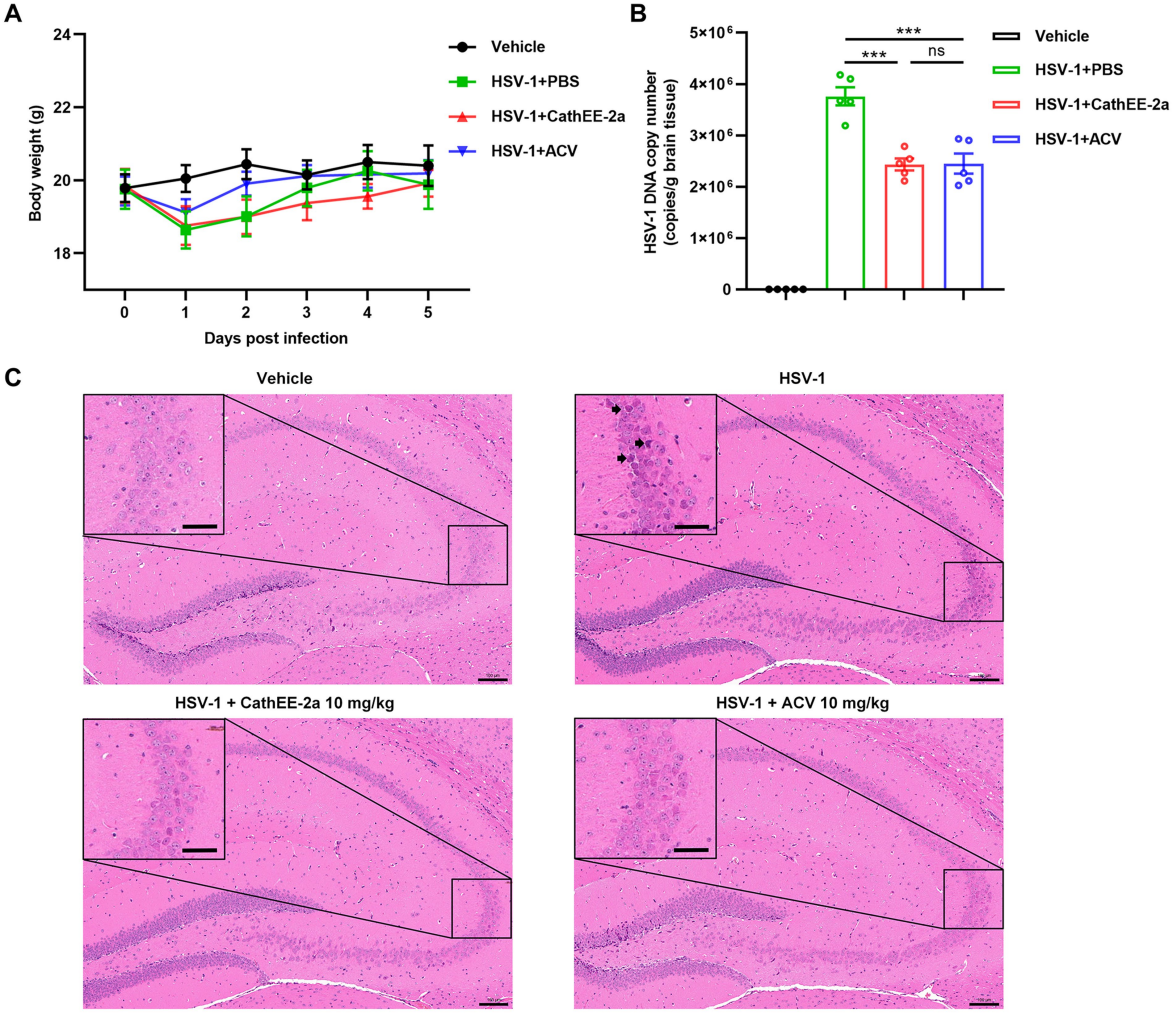
Anti-HSV-1 efficacy of CathEE-2a *in vivo*. **(A)** Body weight monitoring of C57BL/6 J mice (*n* = 5) for 5 days post infection. Mice were intraperitoneally injected daily with 10 mg/kg CathEE-2a, 10 mg/kg ACV, or an equal volume of PBS (vehicle). **(B)** Quantification of HSV-1 viral copies in brain tissues of infected animals. Viral loads were determined by qPCR (*n* = 5). **(C)** H&E staining of brain tissue sections from each group. Black arrows indicate atrophic neurons. Scale bars: original picture, 100 μm; enlarged picture, 25 μm. Statistical significance was determined by one-way ANOVA followed by Tukey’s post-hoc test for multiple pairwise comparisons **(B)**. ***p < 0.001; ns, not significant.

To validate the translational potential of CathEE-2a, we established an HSV-1 infection model via footpad inoculation of WT mice. As shown in Figure 5A, body weight did not differ significantly among the groups, indicating the absence of overt toxicity induced by CathEE-2a treatment. In addition, we measured viral loads in brain tissues, a key target organ of HSV-1, via qPCR. Notably, CathEE-2a significantly reduced brain viral loads at 5 days post infection, with no statistically significant difference observed between the CathEE-2a and ACV groups (Figure 5B). Histopathological analysis of brain tissues using H&E staining revealed that HSV-1 infection triggered marked pathological changes in the hippocampus, including neuronal shrinkage. In contrast, there was less neuronal damage in the hippocampus of the CathEE-2a and ACV treated groups (Figure 5C). Taken together, these results suggest that CathEE-2a can inhibit HSV-1 infection *in vivo* and alleviate neuronal damage in infected mice, highlighting its promising translational potential.

## 4 Discussion

HSV-1 is one of the most prevalent DNA viruses globally, responsible for diseases that range from mild mucocutaneous lesions to severe, life-threatening encephalitis (Sadowski et al., 2021). Owing to its ability to establish lifelong latency and the increasing emergence of drug-resistant strains, current antiviral therapies are unable to achieve complete viral eradication. These limitations highlight the critical need for developing novel and more effective anti-HSV-1 therapeutic strategies (Essa et al., 2022).

Animal-derived ingredients in traditional Chinese medicine represent valuable reservoirs for modern drug discovery. Hedgehog skin, traditionally used to treat inflammatory and hemorrhagic disorders, is one such example (Xiang, 2013). In this study, we showed in U251 cells that HSE inhibits HSV-1 infection in a dose-dependent manner, with the antiviral effect of 50 μg/mL HSE comparable to that of 5 μg/mL ACV, a first-line clinical antiviral agent. This finding expands the conventional therapeutic scope of hedgehog skin beyond anti-inflammatory and hemostatic applications, demonstrating that its extract exhibits direct antiviral activity. Given the increasing clinical burden of acyclovir-resistant HSV-1, the chemical complexity of HSE may also reduce the likelihood of resistance development, positioning it as a promising natural candidate for anti-HSV-1 therapy. However, as wild animal resources continue to decline and hedgehogs are now classified as locally protected species, identifying alternative bioactive components that can replicate the antiviral properties of hedgehog skin is essential for sustainable therapeutic development.

Cathelicidin (CAMP), a key effector molecule of the innate immune system, possesses both direct antiviral activity and broad immunomodulatory functions, making it an attractive scaffold for antiviral drug development (Cebrián et al., 2023). In this study, we found that HSV-1 infection markedly upregulated *CAMP* expression in HSFs, consistent with previous reports showing that influenza viruses and other herpesviruses induce *CAMP* expression in epithelial and fibroblast cells. Beyond supporting this conserved response, our findings highlight the evolutionary preservation of CAMP-mediated antiviral immunity across species, establish CAMP as an intrinsic antiviral defense factor in hedgehog skin, and support its identification as one of the bioactive components of HSE. CathEE—a mature cathelicidin derived from the European hedgehog and identified through NCBI sequence screening—exhibits the characteristic amphipathic *α*-helical structure of antiviral cathelicidins. Its hydrophobic domain interacts with and disrupts the HSV-1 lipid envelope, while its hydrophilic domain engages viral glycoproteins or host receptors to impede viral entry, a mechanism shared with human LL-37 and porcine PR-39 (Holani et al., 2016; Chessa et al., 2022). Functional assays and cytotoxicity evaluations further demonstrated that CathEE significantly reduces viral loads in HSV-1-infected cells without inducing toxicity in U251 cells at the tested concentrations. These properties mitigate the host-cell damage commonly associated with peptide-based therapeutics and underscore the promising translational potential of CathEE as a novel antiviral candidate. Nonetheless, we acknowledge that the relative contribution of CathEE to the overall antiviral activity of whole HSE remains to be experimentally dissected, as we have not performed fractionation of HSE, depletion/neutralization of CAMP from the extract, or direct comparison of extracts with and without the peptide. Thus, we hypothesize that CathEE is one of the active antiviral components contributing to the observed anti-HSV-1 effects of HSE, rather than the sole principal component.

In this study, we aimed to develop a cathelicidin-derived peptide with anti-HSV-1 activity through rational engineering. Starting from the native scaffold, we designed three truncated analogs (CathEE-1 to CathEE-3) and generated two optimized derivatives (CathEE-2a and CathEE-2b) through stepwise sequence refinement. *In vitro* analyses demonstrated that CathEE-2a potently inhibited HSV-1. Moreover, in a murine model of HSV-1 infection, CathEE-2a significantly reduced viral burden in the brain—an observation of particular translational importance, as HSV-1 encephalitis carries high mortality and current frontline antivirals show limited therapeutic benefit due to poor blood–brain barrier (BBB) permeability. While further studies are warranted, these results suggest that CathEE-2a may exert activity within the central nervous system (CNS), either through BBB penetration or by acting directly at sites of CNS infection. Consistent with the viral load data, histopathological evaluation confirmed that CathEE-2a mitigated HSV-1-induced brain tissue damage. Together, these findings support a role for host immune modulation in mediating the observed reductions in brain viral load and tissue pathology. We therefore sought to define the mechanism underlying this immunomodulatory effect.

Our mechanistic analyses revealed that the antiviral activity of CathEE-2a is largely mediated through modulation of host immune responses, with a critical dependence on the type I IFN signaling pathway. Functional analyses further revealed that CathEE-2a targets the post-entry stage of infection, thereby localizing its antiviral activity to the mid-to-late phases of the viral life cycle. This temporal specificity indicates that CathEE-2a is unlikely to act on the viral particle or cellular attachment receptors, but instead affects viral or host factors required for viral genome replication, transcription, or assembly. Importantly, this antiviral effect is contingent upon an intact type I IFN response. At the molecular level, CathEE-2a promotes the upregulation of type I IFNs and their downstream effector genes, thereby reinforcing the host antiviral state. Specifically, it activates innate immune signaling pathways, enhances the secretion of IFN-*α*/*β*, and induces the expression of antiviral genes including *Isg15* and *Mx1*, collectively establishing a robust intracellular antiviral environment. Unlike conventional therapeutics that primarily target viral components, CathEE-2a modulates host immune pathways, a strategy that markedly reduces the likelihood of resistance development (Aribi Al-Zoobaee et al., 2020; Stoyanova et al., 2023). For HSV-1 to overcome such host-targeted inhibition, multiple adaptive mutations affecting host-virus interactions would be required, representing a substantially higher evolutionary barrier than the single-site mutations often sufficient to confer resistance to traditional antivirals.

While the present study establishes both the antiviral activity and mechanistic basis of CathEE-2a, several key questions remain regarding its therapeutic potential. The pharmacodynamic profile, including detailed dose–response relationships and temporal kinetics, has yet to be fully characterized. In addition, its effects on survival and neurological outcomes, as well as its pharmacokinetic properties and ability to penetrate the blood–brain barrier, warrant further investigation in future studies.

To date, most natural antiviral peptides have been identified from insects, plants, or mammalian blood and mucosal tissues (Agerberth et al., 1996; Méndez-Samperio, 2010; Feng et al., 2020; Omer et al., 2025). Here, we report a peptide with potent anti-HSV-1 activity derived from HSE for the first time. This discovery not only expands the known repertoire of natural sources of antiviral peptides but also provides experimental support for the development of sustainable alternatives to rare animal-derived medicinal resources.

## Data Availability

The original contributions presented in the study are included in the article/Supplementary material, further inquiries can be directed to the corresponding authors.

## Footnotes

1
https://alphafoldserver.com/

2
https://heliquest.ipmc.cnrs.fr
