# Correction: A hedgehog cathelicidin-derived peptide exhibits antiviral activity against herpes simplex virus type 1 infection

**DOI:** 10.3389/fmicb.2026.1825082

**Published:** 2026-03-23

**Authors:** Enjie Deng, Yaping Pei, Kun Yuan, Juan Yang, Suncheng-ai Cao, Xinyan Yang, Guilan Li, Libin Liang, Lin Jin, Tengyu Zhu

**Affiliations:** 1 Shanxi Key Laboratory for Modernization of TCVM, College of Veterinary Medicine, Shanxi Agricultural University, Taiyuan, China; 2 Department of Anesthesiology, First Affiliated Hospital of Kunming Medical University, Kunming, China

**Keywords:** antiviral activity, CathEE-2a, drug candidate, hedgehog cathelicidin, HSV-1, type I interferons

In the published article, there was an error in the **Funding** statement. The grant numbers for the National Natural Science Foundation of China were listed in an incorrect order.

The **Funding** statement previously stated:

“The author(s) declared that financial support was received for this work and/or its publication. This project was supported by the National Natural Science Foundation of China (Grants No. 32322014, 32460138, and 32570613), the Distinguished and Excellent Young Scholar Cultivation Project of Shanxi Agricultural University (Grant No. 2022JQPYGC03), and the Funds for International Cooperation and Exchange of the Science and Technology Department of Shanxi Province (Grant No. 202404041101012).”

The correct **Funding** statement is:

“The author(s) declared that financial support was received for this work and/or its publication. This project was supported by the National Natural Science Foundation of China (Grants No. 32570613, 32322014, and 32460138), the Distinguished and Excellent Young Scholar Cultivation Project of Shanxi Agricultural University (Grant No. 2022JQPYGC03), and the Funds for International Cooperation and Exchange of the Science and Technology Department of Shanxi Province (Grant No. 202404041101012).”

In the published article, there were errors in the **Supplementary material**.

1. The first **Supplementary figure** was incorrectly labeled as “Supplementary Figure 2”. The correct label should be “**Supplementary Figure 1**”.

2. In Supplementary Figure 2B, the step box labeled “37 °C, 24 h qPCR analysis” contained incorrect text. The correct text is “qPCR analysis”.

There was a mistake in Figure 5 as published. The peptide name was incorrectly labeled as “CathEA-2a” in Figure 5C. The correct label should be “CathEE-2a”. The corrected Figure 5 appears below.

**Figure 5:**
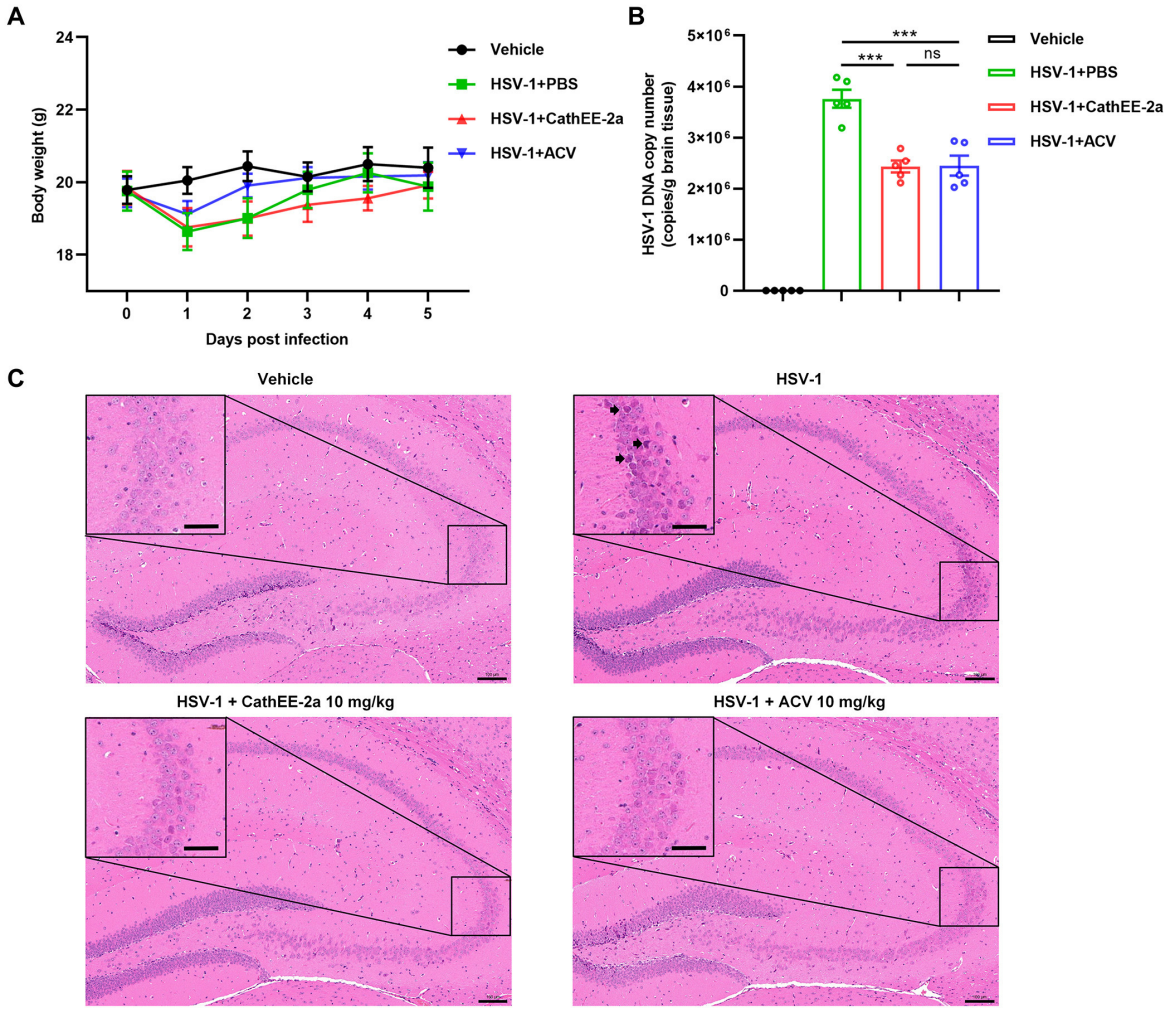
Anti-HSV-1 efficacy of CathEE-2a *in vivo*. **(A)** Body weight monitoring of C57BL/6J mice (*n* = 5) for 5 days post-infection. Mice were intraperitoneally injected daily with 10 mg/kg CathEE-2a, 10 mg/kg ACV, or an equal volume of PBS (vehicle). **(B)** Quantification of HSV-1 viral copies in brain tissues of infected animals. Viral loads were determined by qPCR (*n* = 5). **(C)** H&E staining of brain tissue sections from each group. Black arrows indicate atrophic neurons. Scale bars: original picture, 100 μm; enlarged picture, 25 μm. Statistical significance was determined by one-way ANOVA followed by Tukey's post-hoc test for multiple pairwise comparisons **(B)**. ^***^p < 0.001; ns, not significant.

The authors apologize for these errors and state that they do not change the scientific conclusions of the article in any way.

The original article has been updated.

