# Supplementary material for: A hedgehog cathelicidin-derived peptide exhibits antiviral activity against herpes simplex virus type 1 infection
Source: Front Microbiol. 2026 Feb 18;17:1770133. doi: 10.3389/fmicb.2026.1770133 (PMC12957079; doi:10.3389/fmicb.2026.1770133)
Supplement: Supplementary file 1 [file Data_Sheet_1.PDF]

Supplementary Material

1 Supplementary Tables

Supplementary Table 1. The amino acid sequences and the physicochemical properties of template peptide CathEE and its modified peptides.

Physicochemical Property	CathEE	CathEE-1	CathEE-2	CathEE-3	CathEE-2a	CathEE-2b
Sequence	DLIKKGTQK IGRKLRKVG QQIKDFIRN	IKKGTQKIG RKLRKVGQ QIKDFIR	IGRKLRKVG QQIKDFIRN	LIKKGTQKI GRKLRKVG Q	IVKRLRKVV KKIVKFIKK	RIKKVVKKII KKLKKVVR
Length	27	24	18	18	18	18
Charge	+7	+8	+5	+7	+9	+10
Molecular weight (Da)	3181.82	2839.47	2169.60	2051.55	2223.95	2204.95
Isoelectric point	11.17	11.76	11.73	12.04	12.05	12.06
Molecular formula	$C_{141}H_{250}N_{46}O_{37}$	$C_{127}H_{228}N_{42}O_{31}$	$C_{96}H_{169}N_{33}O_{24}$	$C_{91}H_{171}N_{31}O_{22}$	$C_{107}H_{199}N_{31}O_{19}$	$C_{104}H_{202}N_{32}O_{19}$
Hydrophilia	Hydrophilic	Hydrophilic	Hydrophilic	Hydrophilic	hydrophobic	Hydrophilic
Stability	stable	stable	stable	stable	stable	stable
Subcellular localization	Cytoplasm	Cytoplasm	Cytoplasm	Cytoplasm	Cytoplasm	Cytoplasm

Supplementary Table 2. The primer sequences.

Gene	Species	Primer Sequences (5′→3′)
UL30	*Human herpesvirus 1*	F: TTCGAGATGCTGTTGGCCTT<br>R: GAAGGGCCAGTCGAAGTTGA
18S rDNA	*Homo sapiens*	F: CGGCTACCACATCCAAGGAA<br>R: GCTGGAATTACCGCGGCT
18S rDNA	*Mus musculus*	F: GTAACCCGTTGAACCCCATT<br>R: CCATCCAATCGGTAGTAGCG
CAMP	*Erinaceus europaeus*	F: GTGGTTACCGTTGCTACTGCTG<br>R: GGAACTGCCTCGTTGTCTTGG
A-tubulin	*Erinaceus europaeus*	F: GCGTGCCTTTGTTCATTGGT<br>R: TCCCTGTAAAAGCAGCACCT
IFNA	*Homo sapiens*	F: CTCATACACCAGGTCACGCT<br>R: AGTGTAAAGGTGCACATGACG
IFNB	*Homo sapiens*	F: AGCTGAAGCAGTTCCAGAAG<br>R: AGTCTCATTCCAGCCAGTGC
MX1	*Homo sapiens*	F: GTTTCCGAAGTGGACATCGCA<br>R: CTGCACAGGTTGTTCTCAGC
ISG15	*Homo sapiens*	F: CGCAGATCACCCAGAAGATCG<br>R: TTCGTCGCATTTGTCCACCA
HPRT	*Homo sapiens*	F: GCTATAAATTCTTTGCTGACCTGCTG<br>R: AATTACTTTTATGTCCCCTGTTGACTGG
Ifna	*Mus musculus*	F: ATGGCTAGGCTCAGCACTTTC<br>R: CTCACTCAGACTTGCCAGCA
Ifnb	*Mus musculus*	F: GCCTTTGCCATCCAAGAGATGC<br>R: ACACTGTCTGCTGGTGGAGTTC
Isg15	*Mus musculus*	F: TGACTGTGAGAGCAAGCAGC<br>R TGACTGTGAGAGCAAGCAGC
Mx1	*Mus musculus*	F: GACCATAGGGGTCTTGACCAA<br>R: AGACTTGCTCTTTCTGAAAAGCC
Hprt	*Mus musculus*	F: CTCATGGACTGATTATGGACAGGAC<br>R: GCAGGTCAGCAAAGAACTTATAGCC

2 Supplementary Figure

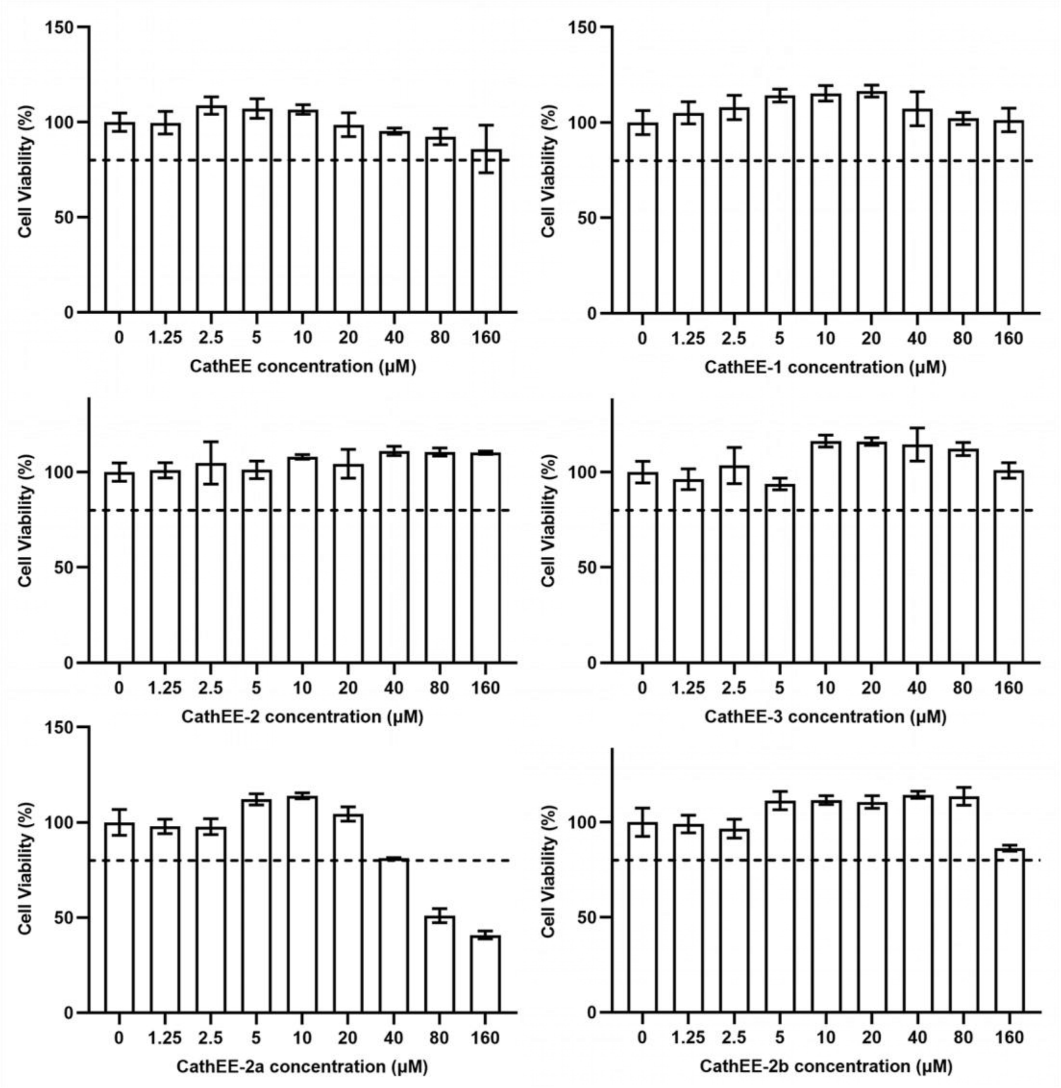

Supplementary Figure 1. The cytotoxicity of cathelicidin CathEE and its modified peptides on U251 cells. In the figure, peptide concentrations at which cell viability exceeded 80% were defined as exhibiting no significant cytotoxicity. Data are from 3 independent experiments and are presented as mean ± SEM.

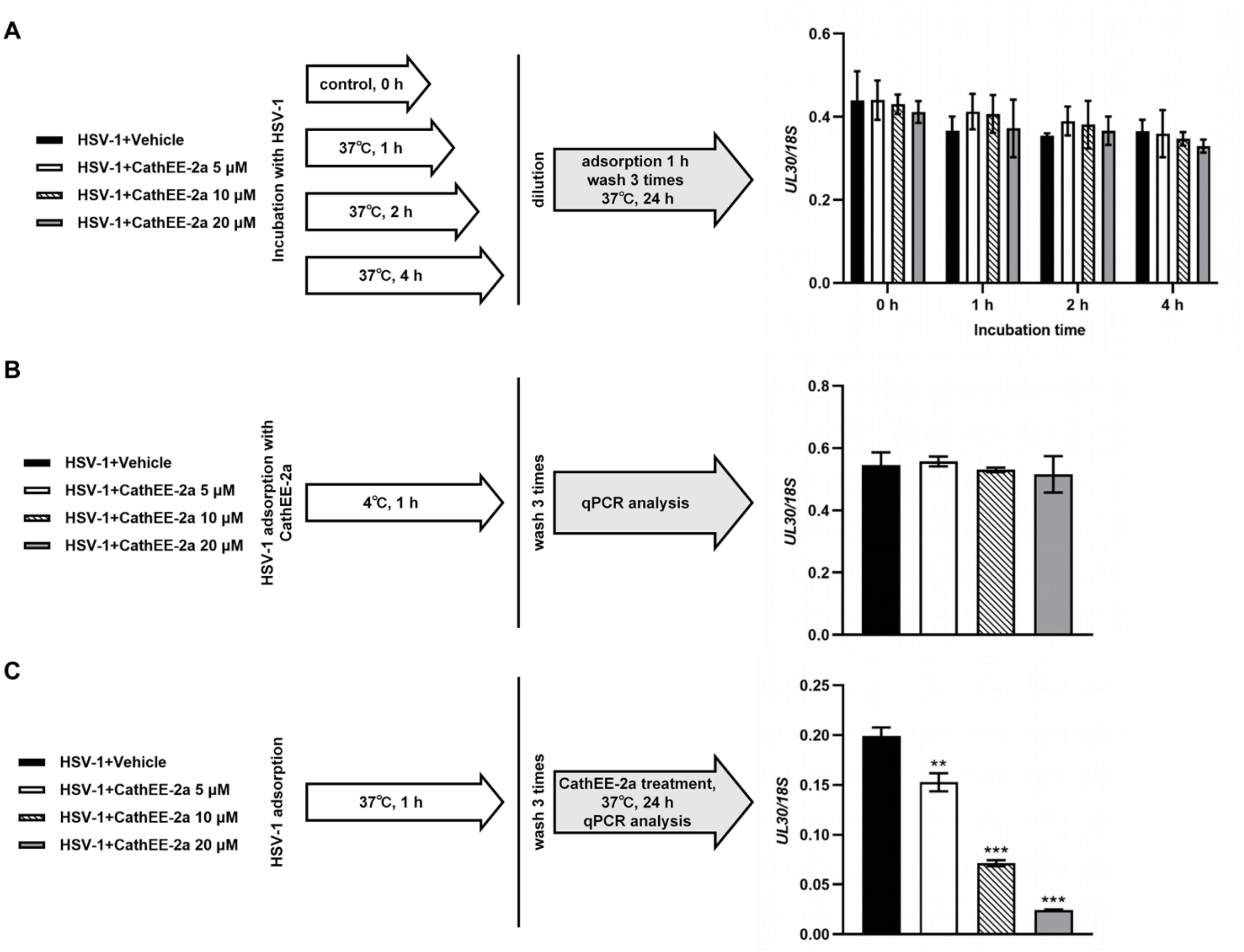

Supplementary Figure 2. CathEE-2a inhibits HSV-1 infection at the post-entry stage. **(A)** Virucidal assay. HSV-1 particles (2×10^8 PFU/mL) were incubated with the indicated concentrations of CathEE-2a at 37 °C for 1, 2, or 4 h, followed by 200-fold dilution to terminate peptide activity. Viral replication was assessed by qPCR analysis of the HSV-1 *UL30* gene in infected U251 cells, with *UL30* levels normalized to the *18S rDNA* reference gene. **(B)** Adsorption assay. U251 cells were incubated with HSV-1 (MOI = 0.05) in the presence or absence of CathEE-2a at 4 °C for 1 h. After removal of unbound virus and peptide by washing, cell-associated HSV-1 *UL30* levels were determined by qPCR and normalized to *18S rDNA*. **(C)** Post-entry assay. U251 cells were infected with HSV-1 at 37 °C for 1 h to allow viral entry, washed to remove unbound virus, and subsequently treated with CathEE-2a for 24 h. Intracellular HSV-1 *UL30* gene levels were quantified by qPCR using *18S rDNA* as the internal reference. Data are from 3 independent experiments and are presented as mean ± SEM. Statistical significance was determined by two-way ANOVA with Tukey's post-hoc test **(A)**, or one-way ANOVA with Tukey's post-hoc test **(B, C)**. $^{**}p < 0.01$; $^{***}p < 0.001$.

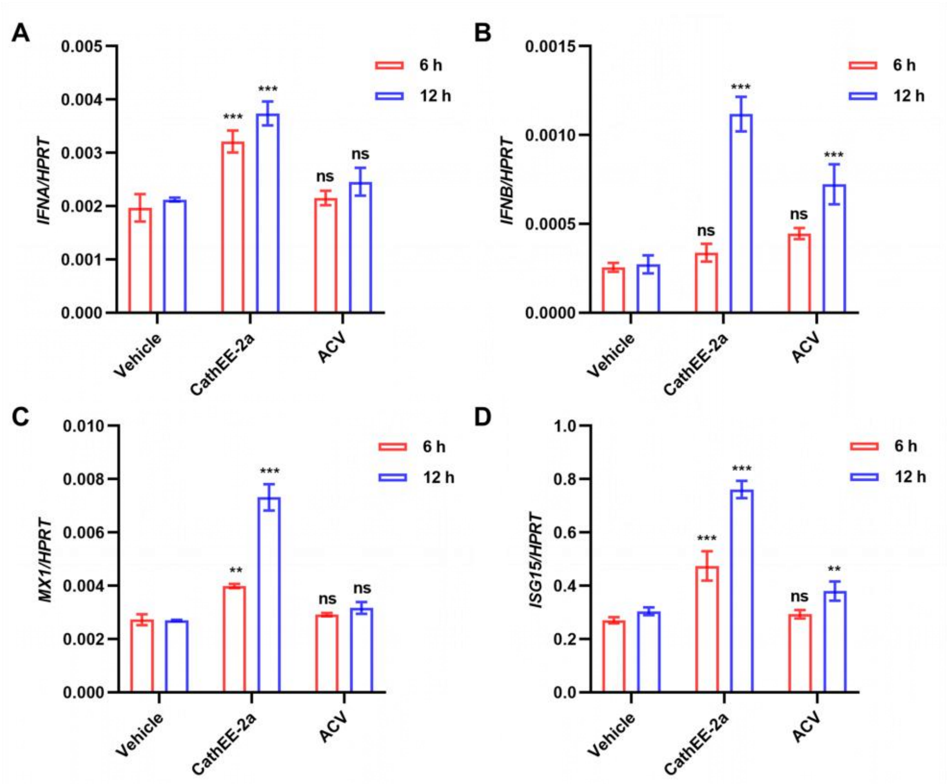

Supplementary Figure 3. CathEE-2a activates the type I interferon signaling pathway in HSV-1-infected U251 cells. qPCR analysis of type I IFN (*IFNA/IFNB*) and its downstream effector genes *ISG15* and *MX1* in U251 cells treated with 10 μM CathEE-2a or 10 μM ACV at 6 or 12 h. Data are from 3 independent experiments and are presented as the mean ± SEM. Statistical significance was determined via two-way ANOVA by Tukey's post-hoc test for multiple pairwise comparisons. **$p < 0.01$; ***$p < 0.001$; ns, not significant.
